# Monitoring food pathogens: Novel instrumentation for cassette PCR testing

**DOI:** 10.1371/journal.pone.0197100

**Published:** 2018-05-10

**Authors:** Darin Hunt, Curtis Figley, Dammika P. Manage, Jana Lauzon, Rachel Figley, Linda M. Pilarski, Lynn M. McMullen, Patrick M. Pilarski

**Affiliations:** 1 CBF Systems Inc., College Plaza, Edmonton, AB, Canada; 2 Department of Oncology, University of Alberta and Cross Cancer Institute, Edmonton, AB, Canada; 3 Department of Agricultural, Food and Nutritional Science, University of Alberta, Edmonton, AB, Canada; 4 Division of Physical Medicine & Rehabilitation, Department of Medicine, University of Alberta, 5–005 Katz Group Centre for Pharmacy and Health Research, Edmonton, AB, Canada; University of Helsinki, FINLAND

## Abstract

In this manuscript, we report the design and development of a fast, reliable instrument to run gel-based cassette polymerase chain reactions (PCR). Here termed the GelCycler Mark II, our instrument is a miniaturized molecular testing system that is fast, low cost and sensitive. Cassette PCR utilizes capillary reaction units that carry all reagents needed for PCR, including primers and Taq polymerase, except the sample, which is loaded at the time of testing. Cassette PCR carries out real time quantitative PCR followed by melt curve analysis (MCA) to verify amplicon identity at the expected melt temperature (T_m_). The cassette PCR technology is well developed, particularly for detecting pathogens, and has been rigorously validated for detecting pathogenic *Escherichia coli* in meat samples. However, the work has been hindered by the lack of a robust and stable instrument to carry out the PCR, which requires fast and accurate temperature regulation, improved light delivery and fluorescent recording, and faster PCR reactions that maintain a high sensitivity of detection. Here, we report design and testing of a new instrument to address these shortcomings and to enable standardized testing by cassette PCR and commercial manufacture of a robust and accurate instrument that can be mass produced to deliver consistent performance. As a corollary to our new instrument development, we also report the use of an improved design approach using a machined aluminum cassette to meet the new instrument standards, prevent any light bleed across different trenches in each cassette, and allow testing of a larger number of samples for more targets in a single run. The GelCycler Mark II can detect and report *E*. *coli* contamination in 41 minutes. Sample positives are defined in as having a melt curve comparable to the internal positive control, with peak height exceeding that of the internal negative control. In a fractional analysis, as little as 1 bacterium per capillary reaction unit is directly detectable, with no enrichment step, in 35 cycles of PCR/MCA, in a total time of 53 minutes, making this instrument and technology among the very best for speed and sensitivity in screening food for pathogenic contamination.

## Introduction

Food safety is becoming increasingly an urgent worldwide priority, with frequent reports of pathogen-mediated disease outbreaks [1–3]. *E*. *coli*, *Salmonella enterica*, *Campylobacter jejuni* and *Listeria monocytogenes* infections have worldwide importance. Rapid on-site molecular testing is urgently needed as products are processed. Although a variety of methods are available to screen for foodborne pathogens, most are relatively slow, requiring 24–48 hours for results and necessitating frequent food recalls if pathogens are detected. There are currently many approaches to characterizing pathogenic bacteria, including Shiga Toxigenic *E*. *coli*, using a variety of conventional molecular methods including polymerase chain reactions (PCR) [4–6] and immunological methods, for example, immunoassays for E. coli O antigens [7–9] but in general these methods are technically demanding and expensive. Culture methods characteristically take 18 hours or longer from sampling to identification of a presumptive positive sample [9,10], with a further several days required to confirm by the growth of colonies in some cases on selective growth media [11], making inventory control challenging and increasing the risk of large recalls. The meat industry needs a process that can satisfy national and international regulatory requirements and will rapidly identify deviations in processing and/or product that is contaminated with bacterial pathogens so that it can respond accordingly. Pathogen contamination in meat has a very significant economic impact on the meat industry and a serious health impact for the public. The industry needs a fast, sensitive and easy to use molecular method to definitively identify pathogens in food. To facilitate rapid and frequent monitoring of food processing to screen for pathogens, here, we have employed our cassette PCR technology [12–16] on an improved and reliable miniaturized testing platform and on an improved cassette.

For sensitive testing, technology should be able to detect as few as 1–3 cfu (colony forming units) of pathogenic bacteria per 25-375g of a given food product, as defined by the regulatory agencies. Because most testing platforms cannot detect a single bacterium, enrichment protocols are routinely used in which bacteria obtained from defined amounts of food product are cultured in broth to expand the contaminants to a level that is detectable. Usually the enrichment step takes 12–48 hours, resulting in significant delays in identifying food products harbouring pathogenic contaminants, which means more sensitive technology should provide faster readouts. When contamination is low, e.g. 1-3cfu per 375 grams of ground meat, a fractional analysis is required, with multiple enrichment bags being tested. Regulatory agencies define fractional analysis as a test in which 50% or less of enriched samples from deliberately inoculated food score as positive [17,18]. To determine the limit of detection (LOD) for a given testing platform, a fractional analysis is needed to show the platform is capable of detecting one bacterium. Because serial dilutions to low numbers suffer from considerable error, multiple dilutions are tested to determine which of them reach a limiting dilution of pathogen as defined by the fractional analysis. To mimic this food safety testing, as well as to show that our methods could in fact detect a single unenriched bacterium per capillary reaction unit, we needed to perform a fractional analysis of the diluted cultures, testing multiple high dilutions to ensure we could obtain one or more in which 50% or fewer of the replicate tests were positive.

Cassette PCR performs real time quantitative PCR (qPCR) using accumulation of fluorescent LC Green dye to measure the amplification of the template. The resulting qPCR shows increasing fluorescence as the number of cycles increases, which is standard procedure for qPCR. To verify that this accumulation of double stranded amplicons represents the correct product, a melt curve analysis (MCA) is performed at the end to ensure that product having the anticipated T_m_ is present. This allows exclusion from analysis of any products that are unintended, for example primer dimers. Positive and negative controls for each primer set are always integrated into each cassette, providing internal controls for every cassette PCR run.

In the cassette, PCR occurs in capillaries holding desiccated hydrogel that contains all reagents except the sample to be tested. The platform is readily adapted to any number and type of targets, accepting single or multiple samples on the same cassette, with a flexible number of targets and integrated quality controls [12–16]. Cassette PCRs had been performed with unprocessed or minimally processed samples such as buccal swabs, genital swabs and urine on a low cost off the shelf system with the cassette capable of simultaneous testing of 7 samples for up to 4 different targets, including positive and negative quality controls, for only $3.00, or $0.08/test. This design was somewhat flexible to accommodate detection of all required targets, but an improved, manufacturable cassette would be an advantage. In addition, the previous instrumentation was delicate with considerable variability between instruments, subject to the need for intensive calibration to ensure accurate results and required a skilled operator to use the instrument.

Therefore, for the use by the food industry, cassette PCR requires a robust and stable instrument to carry out the PCR with fast and accurate temperature regulation, improvements in light delivery and fluorescent recording during the PCR and MCA, ideally performing faster PCR reactions that still maintain a high sensitivity of detection. It is also important to have an instrument that can eventually be mass-produced with identical performance by every instrument. Hence fast, accurate and reliable instrumentation is required to realize the full potential of this technology. The availability of stable, robust instrumentation is also essential for successful commercialization of cassette PCR. In this work, we report the design and development of such an instrument, here termed the GelCycler Mark II, to reflect its ability to run the gel-based cassette PCR. As a corollary, we have also designed and used a machined aluminum cassette to meet our new instrument standards. Our goal was to confirm this testing platform with an LOD of 1-3cfu by testing of pathogens grown in broth, subjected to serial dilution and tested at high or low concentrations.

The GelCycler Mark II described here, and an improved cassette, requires minimal capital equipment, can be used in the abattoir for detecting food pathogens by existing staff during one work shift from swab to results, and provides results in under an hour using automated data analysis software. It avoids a need for specially trained staff or involvement of a third party laboratory, though its use is also suitable for specialized testing laboratories. With cassette PCR, a food sample can be rapidly processed to confirm its safety before shipping to avoid food recalls. Analysis software self-validates the cassette reactions within a window defined by the position of the positive control and reports only the fluorescence that exceeds the negative control for eventual use on site at food processing facilities.

We show that the GelCycler Mark II can detect and report *E*. *coli* contamination in 41 minutes. Without any enrichment step, as little as a calculated number of 1–3 cfu (approximately 1–3 cfu/6μl) delivered into each capillary reaction unit, as defined by a fractional analysis, is detectable in 35 cycles of PCR/MCA, in a total time of 53 minutes, showing that this new instrument has greater sensitivity and faster reaction times than previous instruments, and appears to rank among the best available for pathogen screening of food products.

### Description of cassette PCR

The basis for the cassette PCR testing strategy is the analysis of genetic material in a discrete sub-microliter plug of hydrogel matrix. Each reaction unit holds a desiccated plug of hydrogel containing PCR reagents and a specific set of primers, with arrays of such reaction units encased in a wax support to make a cassette the size of a large postage stamp. Each capillary reaction unit holds only one set of primers, with multiple reaction units to simultaneously amplify multiple targets in parallel. With all PCR reagents and the LC Green dye polymerized into the hydrogel, the user need only deliver unprocessed sample to the delivery port. By incorporating the components of the PCR reaction mixture within the cross-linked hydrogel plug, amplification and product detection occur *in situ*, simplifying assay readouts and avoiding the need for micro valves or pumps that complicate manufacturing. Cassettes holding desiccated reaction units can be stored for at least 7 months at refrigerator or freezer temperatures or 3 months at room temperature [13], without loss of enzyme/reagent function. A significant innovation is the capability for detection of multiple targets in separate reaction units on the same cassette, enabling the creation of cassette test panels to screen for suites of pathogens or multiple markers for a given pathogen [12–16], in this case to monitor food safety. An assembly of reaction units within each cassette can simultaneously perform multiple independent PCR reactions incorporating different primer sets in each reaction unit, allowing simultaneous tests and controls on each sample, or the same set of tests on samples from multiple different individuals. There is no cross-contamination among adjacent reaction units in the same cassette as the melted wax between capillaries acts as a barrier during the PCR as well as providing a vapor barrier [13–15]. The cassette assembly carries out real time quantitative PCR followed by MCA to verify amplicon identity at the expected T_m_ [15]. Detection is via the binding of the dye LC green that fluoresces when it binds to double stranded DNA, thereby providing a measure of the extent of amplification over time and in the MCA.

### Instrument development

Our previous method of thermocycling placed the sample containing cassette on a single thermal block that was heated and cooled by a Peltier device [12–16,19,20]. The entire block and cassette had to be heated and cooled across the large PCR temperature range for each cycle. The resulting temperature control accuracy, rate of temperature change between PCR steps, and overall cycle times were not as good as desired, and it was not possible to standardize instrument components. Successful commercialization requires an instrument that can be mass produced with identical functionality because all components are standard and their assembly can be reliably reproduced. Here we describe the design for and testing of a novel instrument that can reliably run cassette PCR and which takes advantage of many standardized “off the shelf” electronic and optical components as well as some stringently designed custom components, and an improved PCR cassette.

**Fig 1:**
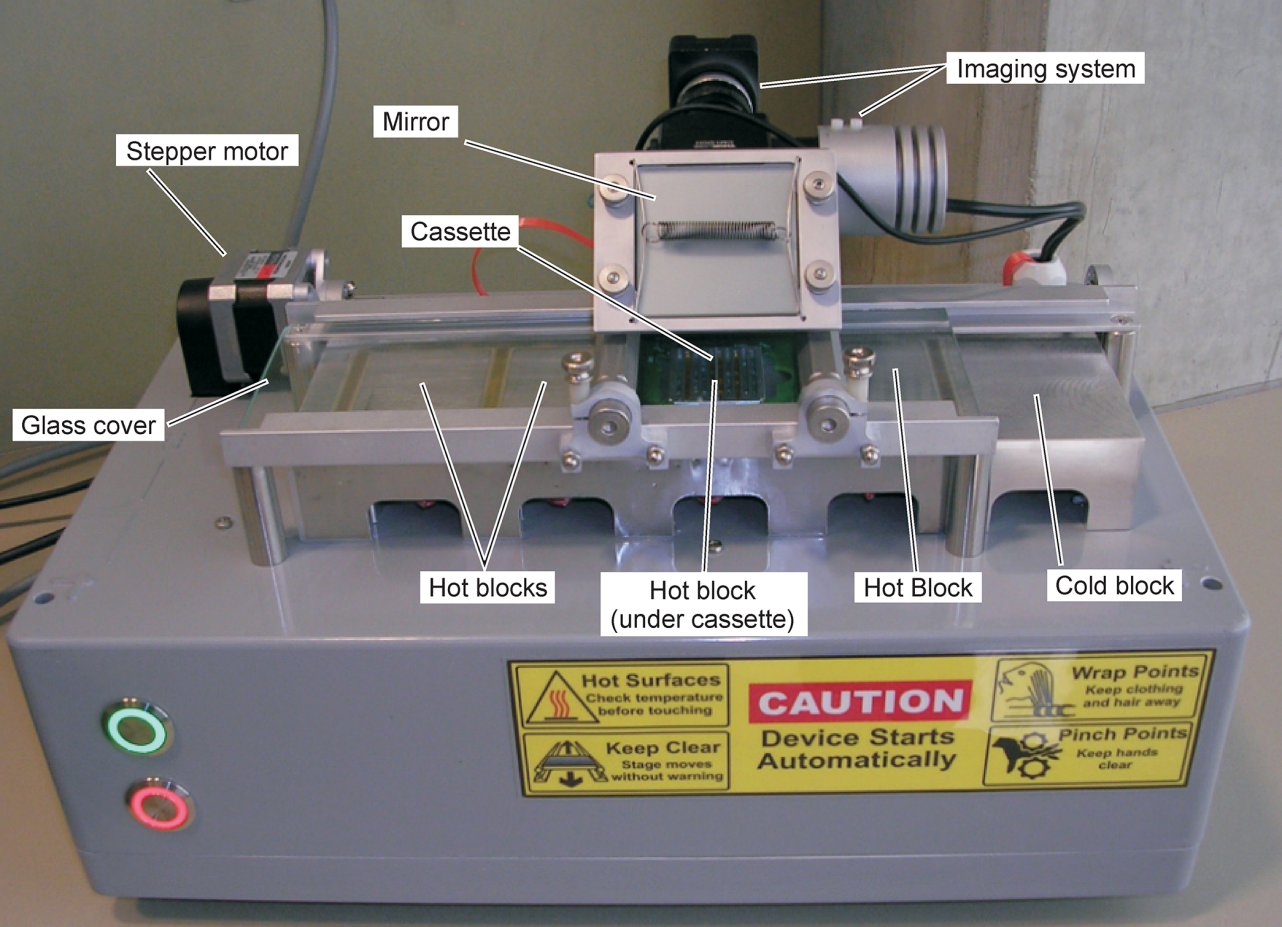
Photograph of the thermocycler device. The device is 40 cm long x 25 cm wide x 23.5 cm tall from the bottom of the enclosure (feet excluded) to the top of the camera system.

The new device, GelCycler Mark II, shown in Fig 1, utilizes an alternate temperature control scheme wherein thermocycling is accomplished by moving the PCR cassette between four temperature controlled hot blocks. The blocks are preheated prior to the start of the PCR run: one is maintained near the anneal temperature, one near the extend temperature and two near the denature temperature. The hot block temperatures are relatively constant except for small variations during PCR and then melt curve data collection, this will be described later. As the cassette is moved to each block in turn, heat flows between the block and cassette; their combined temperatures quickly bringing the cassette to the temperature required for the PCR step performed at that block.

#### Instrument layout and cassette movement

**Fig 2:**
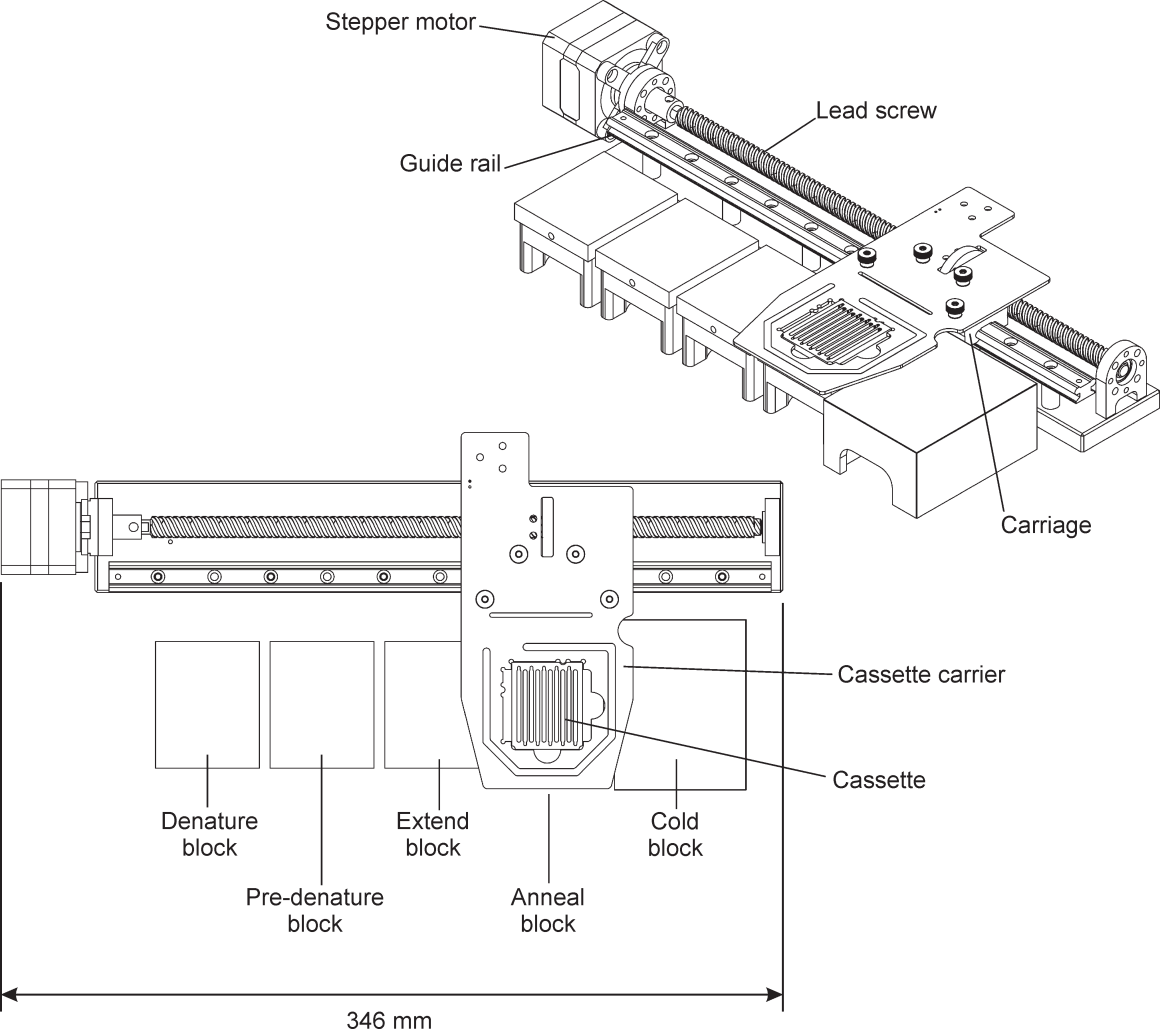
Two views of the hot blocks, cassette, and cassette positioning system. Four hot blocks and a cooling block (on the right) are shown. A stepper motor, lead screw and cassette carrier move the cassette from block to block.

A drawing of the five thermal blocks and the cassette moving mechanism of the GelCycler is shown in Fig 2. The blocks are located side by side in a linear arrangement, but are thermally isolated from one another. As indicated above, there are four hot blocks that are maintained at various temperatures in order to perform PCR at the desired temperatures. A cold block (far right) is used to cool and load/unload the cassette from the instrument. The cassette rests on top of the blocks and a computer controlled stepper motor, lead screw, and cassette carrier move the cassette between the various blocks as required. The cassette is centered on a block when resting there. A glass plate positioned above the hot blocks and cassette reduces convection.

#### Thermal conduction

Both the cassette and hot blocks are made of aluminum, which has a high thermal conductivity, the mating surfaces between the cassette and blocks are manufactured to high flatness, and the contact area between the blocks and the cassette is large so temperature equalization between a block and the cassette is rapid. The addition of a thin layer of mineral oil improves the thermal bond both by drawing the cassette against the hot block due to surface tension and additionally by filling in any small remaining gaps with a coupling material with better heat transfer properties than air. Oil also makes cassette movement smoother and reduces wear on the mating surfaces. When the cassette moves onto a hot block, the cassette and hot block quickly equalize to a temperature between their two initial temperatures. Since the thermal mass of each hot block is more than 10 times greater than the cassette and its contents, the equalization temperature is dominated by the hot block, and variations in the cassette due to such things as the number of capillaries or amount of wax loaded have only a minor effect. The equalization temperature is less than two degrees from the temperature of the hot block before the cassette arrives, as described later.

#### Temperature control

**Fig 3:**
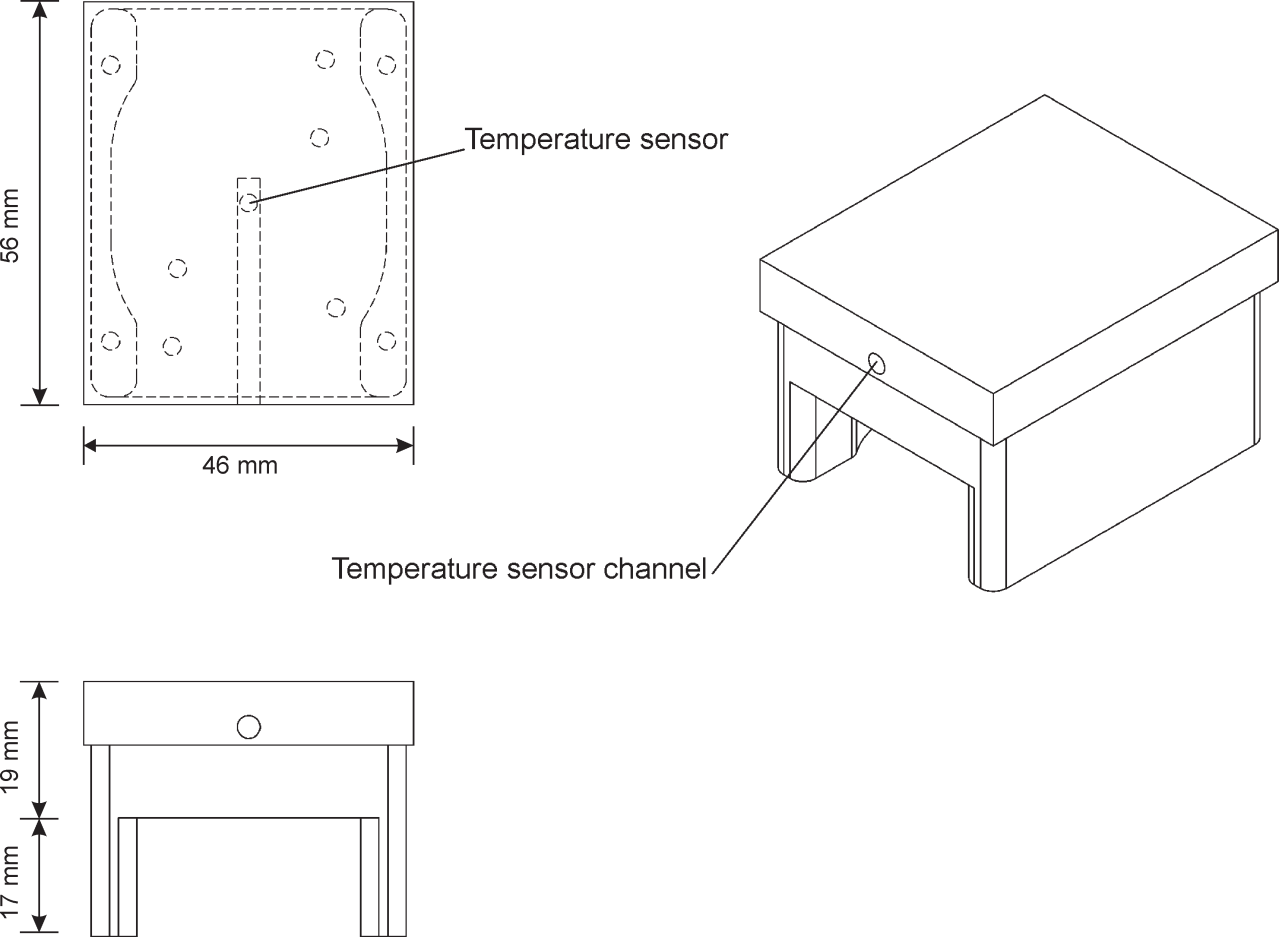
Single hot block with temperature sensor.

**Fig 4:**
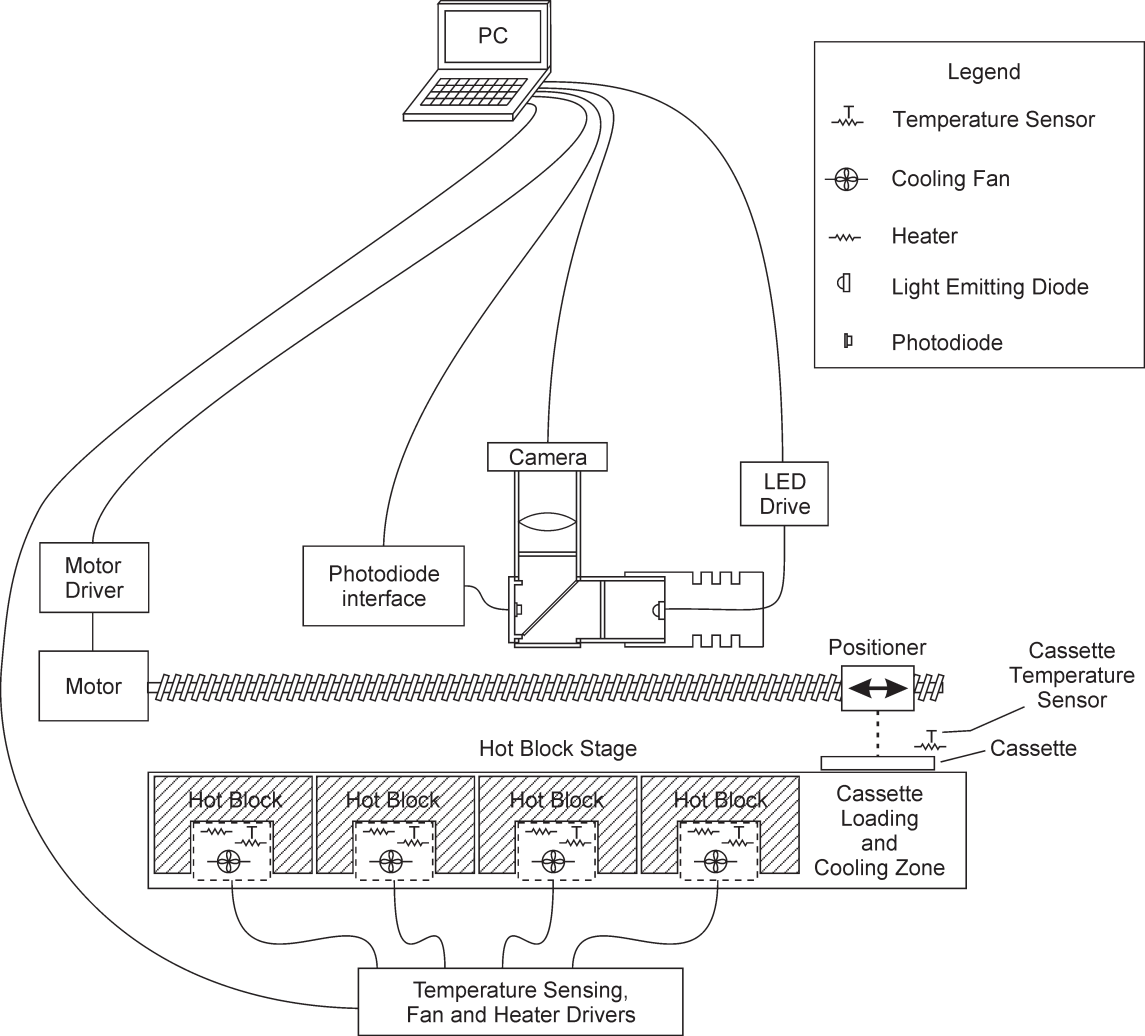
Schematic representation of the thermocycler system.

The temperature of each hot block is independently controlled by a feedback control circuit. A schematic of a single hot block is shown in Fig 3. The block temperature is measured by a resistive temperature detector (RTD) located inside the block near its top surface. A resistive heating element on the bottom and fan below provide heating and cooling respectively. The feedback control algorithm runs on a PC, which is connected to the RTD, heater, and fan by input/output circuitry (Fig 4). Any changes to the block temperature caused by the temperature control circuitry do not need to be fast.

#### Cassette

**Fig 5:**
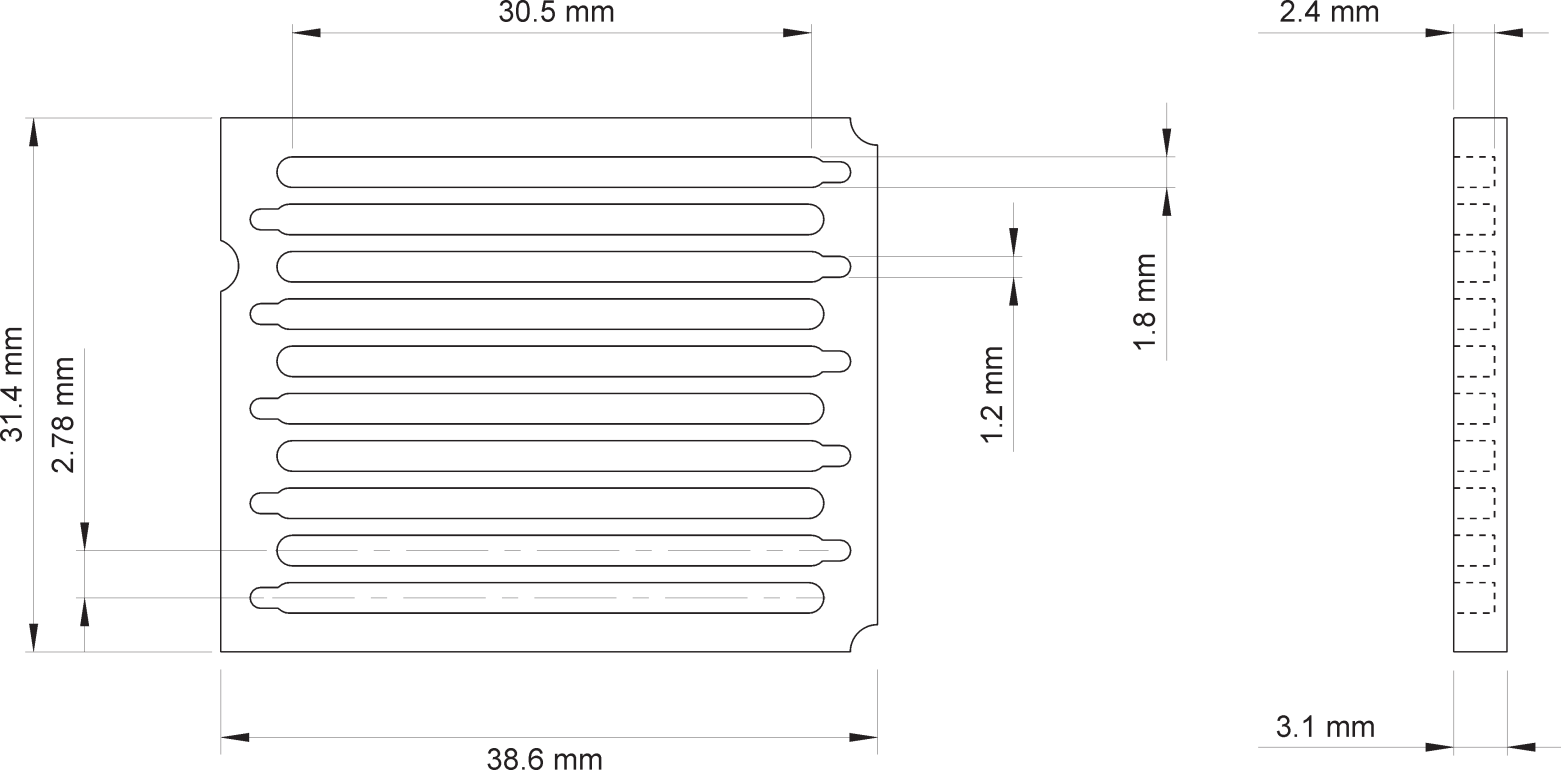
Drawing of the PCR cassette. It is machined from a single piece of aluminum and has 10 trenches (grooves) for holding sample containing capillaries.

**Fig 6:**
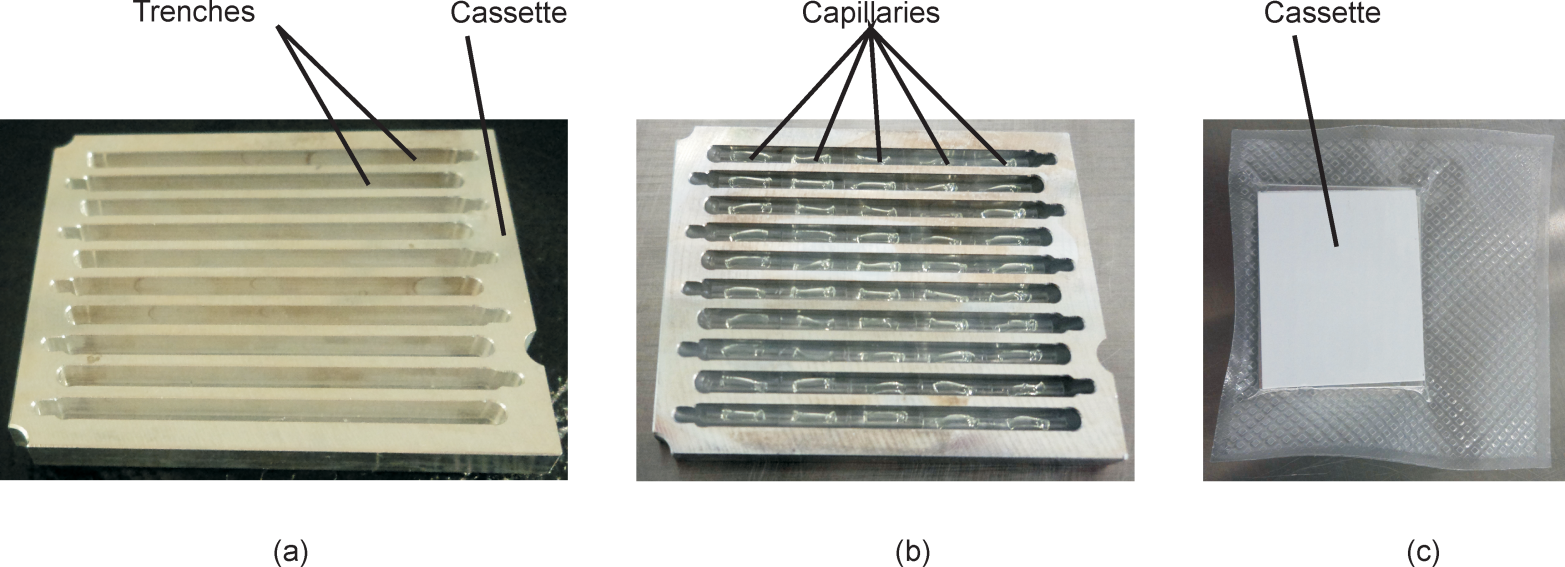
Three views of a PCR cassette. (a) an empty cassette, (b) a cassette loaded with 50 capillaries, and (c) a cassette packaged for storage.

The cassette used with the GelCycler Mark II is computer numerical control (CNC) machined from a single piece of aluminum (Fig 5). Ten trenches are machined into the cassette (Figs 5 and 6(A)) to hold the capillaries. Each channel can hold up to 5 capillaries for a total of 50 capillaries in a cassette (Fig 6(B)) hence it is capable of doing 50 individual PCR reactions. The cassette is designed to quickly and evenly distribute heat to all the capillaries. It has a large, flat surface to give good thermal coupling to the hot blocks. Its short height promotes rapid heat flow in the direction perpendicular to the block surface. The high thermal conductivity aluminum walls conduct heat along the sides of the capillaries so that heat can flow to the capillaries from the sides, not just the bottom. The trenches are only slightly larger than the diameter of the capillaries (capillaries are 1.6 mm in diameter), so the distance of the capillaries from the aluminum walls is small, which helps reduce thermal resistance between the walls and the capillaries. Because the walls are opaque, they also block fluorescence generated in one channel from directly bleeding to adjacent trenches.

**Fig 7:**
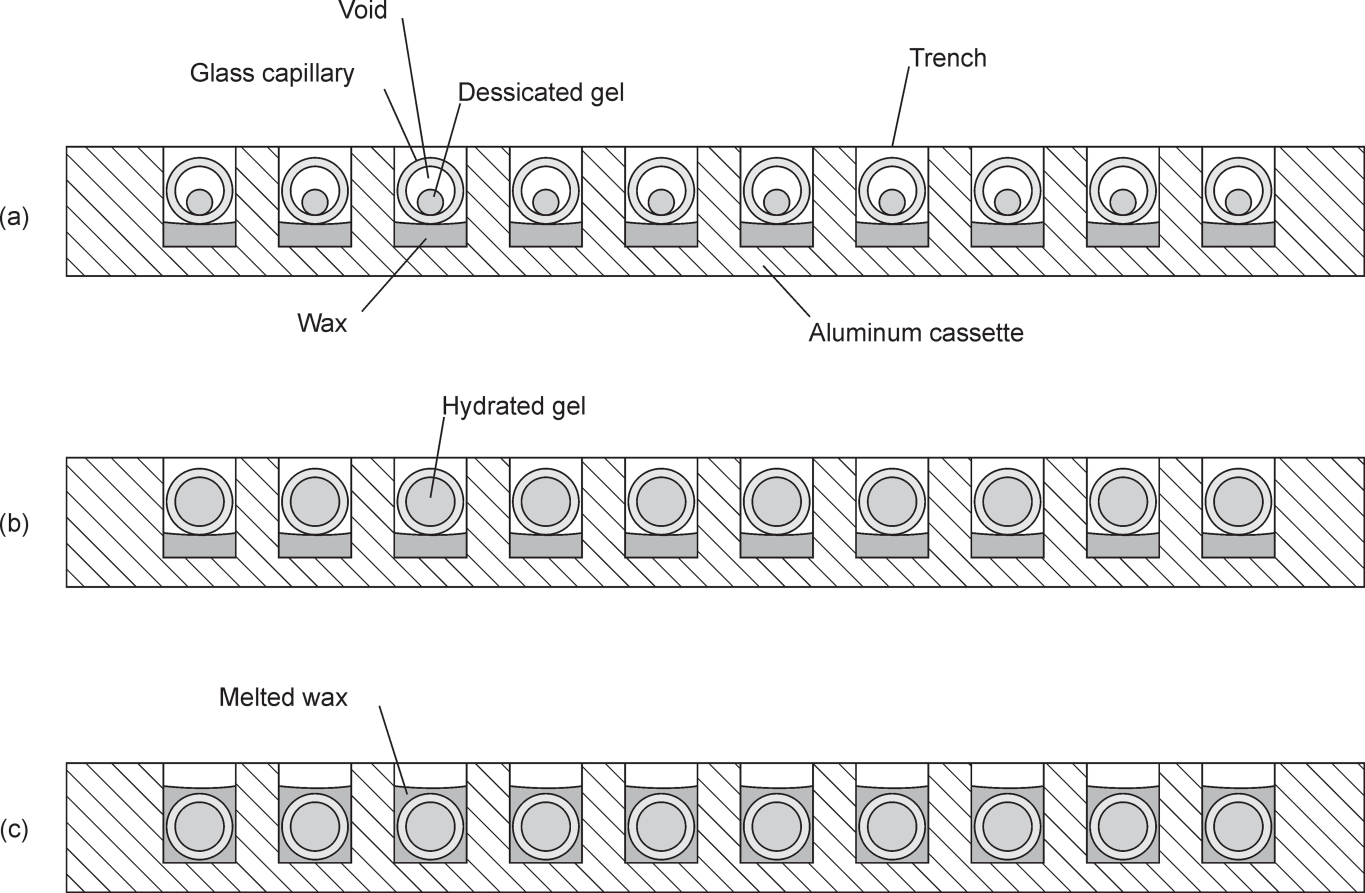
Cross-sectional view of the PCR cassette with capillaries and wax. (a) capillaries where the gel is desiccated before the sample is added. (b) capillaries after the sample has been added and the gel has hydrated, but before the wax is melted. (c) capillaries after the wax has melted after the cassette was heated during the PCR.

When the capillaries are first placed in the cassette trenches (preparation of the capillaries is explained later in the paper), the gel is desiccated inside the capillary forming a noodle shape with the remainder of the internal space filled with air [15] (Fig 7(A)). The sample is administered to the last capillary in a given channel, letting the sample flow through the entire trench by capillary forces thereby hydrating the gels. The sample permeates into the gel, which expands to fill the inside of the capillaries (Fig 7(B)). The capillaries rest on top of the wax that has been placed in the bottom of the cassette trenches during the preparation of the cassette. Once the PCR starts, the wax melts, allowing the capillaries to sink to the bottom of the trenches (Fig 7(C)). The capillaries are submerged in wax during PCR, which prevents dehydration of the capillaries. Melted wax serves as a vapor barrier and also prevents cross contamination between adjacent capillary reaction units, as confirmed previously [13–15].

#### Imaging system

**Fig 8:**
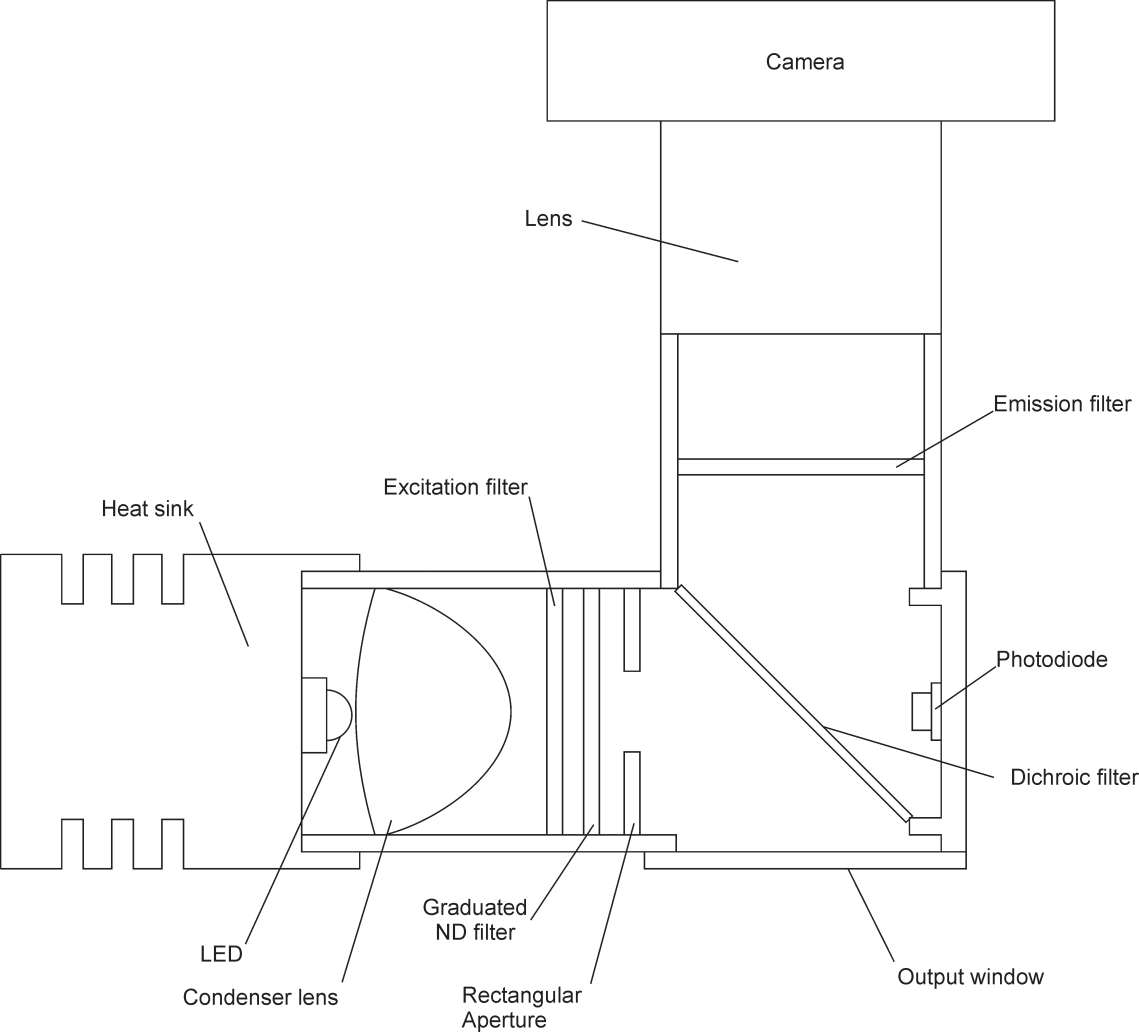
Schematic representation of the epifluorescence system.

The GelCycler includes an epifluorescence camera system to collect fluorescence images. The optical layout of this system is shown in Fig 8. As seen in Fig 1, the camera and these associated optics are located over the extend block. At the end of the extend step of each PCR cycle, the cassette is illuminated and a photograph is taken in order to obtain real-time PCR curves. After PCR is completed with the cassette resting on the extend block, the temperature of the extend block is ramped over the desired temperature range of the melt curve, further photographs of the cassette are acquired during the temperature ramp (typically with 0.2°C intervals). Illumination is provided by a high power LED with a center wavelength of 460 nm. The LED has a beam angle of 100 degrees, so a condenser lens is used to narrow the beam angle and enhance the collimation. A narrower beam angle is required to get a significant amount of the light to the target (the cassette) and for the correct operation of the excitation filter, which is designed for normal incidence light. The lens position relative to the LED was chosen to give an acceptable trade-off between illumination intensity and uniformity at the cassette. A circularly symmetric graduated neutral density (ND) filter improves the uniformity of the light distribution. A rectangular aperture reduces scattered light in the optical system. Most of the LED light is directed toward the cassette by the dichroic filter, but some passes through the dichroic filter to a photodiode, which is used to detect the LED intensity to facilitate adjusting and regulating LED output power accurately. A mirror (Fig 1) bends the light 90° so that the imaging system can be oriented horizontally, reducing the total instrument height. Other components perform a normal function in an epifluorescence system. An opaque lid covers the GelCycler during PCR and melt curve data collection.

Even after passing through the graduated ND filter, the illumination intensity is not as uniform as desired. An illumination calibration and compensation process was developed to increase the accuracy of the fluorescence measurement across the image. A fluorescent reference target was placed at the cassette location, and the target was illuminated by the system. The target was slowly stepped past the imaging system using the cassette carrier while images were periodically recorded. The images were averaged to reduce the effect of any local non-uniformity in the target. A profile of image intensity versus pixel location was thus obtained, which was then used to correct image intensity during normal operation.

The gain correction function is:

$$Gain\;correction\;(Pixel\;location)=\frac{I_{ref}}{I_{ave}(Pixel\;location)}$$

where I_ave_ is the average of the intensities measured for a pixel during calibration and I_ref_ is the maximum pixel intensity in the averaged image during calibration, ignoring the very brightest pixels to reject possible outliers. Corrected intensities are:

$$I_{corrected}(Pixel\;location)=Gain\;correction(Pixel\;location)\times I(Pixel\;location)$$

The calibration process corrects for both illumination and imaging (camera and associated optics) non-uniformity. The effectiveness of the calibration was assessed by imaging a 6 x 11 mm fluorescent target and measuring its intensity with the target moved between the center and four corners of the cassette. The exposure was adjusted to get an image intensity about 80% of full scale. The intensity integrated over the target was constant within +/- 6%, regardless of location.

Two Mark II instruments were made with the components shown below.

- Illumination LED—460 nm LED (LED ENGIN LZ1-10DB00).
- Illumination lens—f = 16.0 mm, AR coated, 25.4 mm diameter, aspheric condenser lens (Thorlabs ACL25416U-A)
- Graduated neutral density filter–custom, circularly symmetric, graduated, neutral density filter. It was printed on a transparency using a laser printer and cut to the required size. The image was a circularly symmetric gray scale gradient with the highest density in the centre.
- Excitation filter—445 nm, 45 nm wide band pass filter (Thorlabs MF445-45).
- Emission filter—510 nm, 42 nm wide band pass filter (Thorlabs MF510-42).
- Dichroic filter—415–470 nm reflection band, 490–720 nm transmission band filter (Thorlabs MD480).
- Output window—50.8 mm diameter, AR coated window (Edmund Optics 48–926).
- Camera lens—12.5 mm, f/1.4 lens (Fujinon HF12.5HA-1B)
- Camera—1288 x 964 pixel, global shutter, monochrome CDD, USB camera (Point Grey CM3-U3-13S2M-CS)
- Photodiode (Opto Diode ODD-5WB)

**Fig 9:**
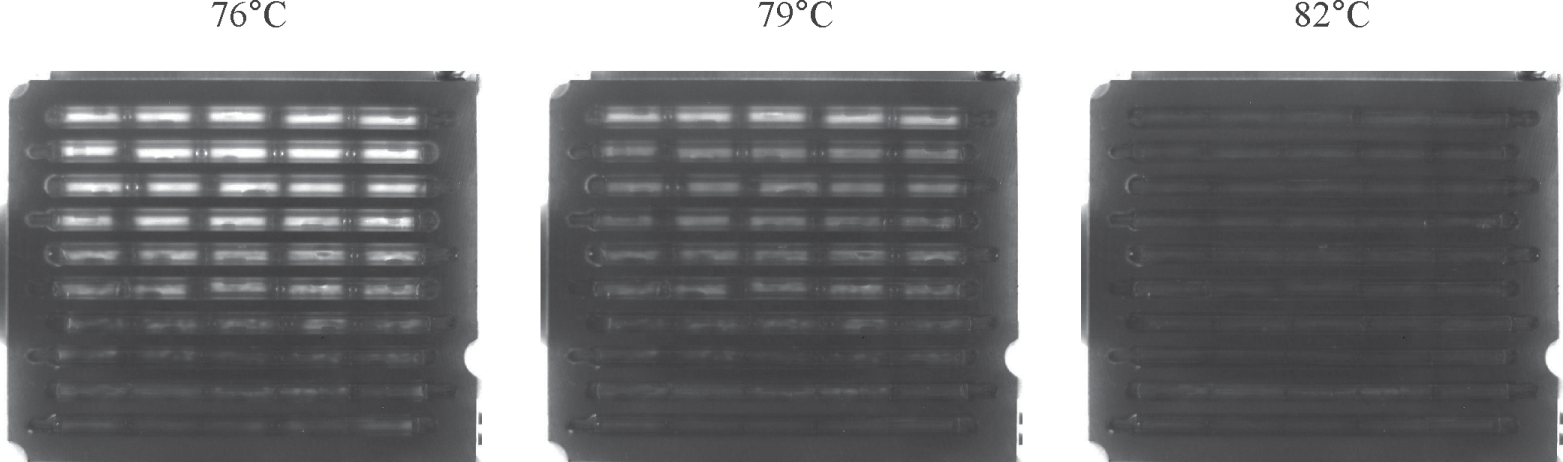
Photographs of the cassette during melt curve data collection. These images were taken during the MCA after 35 cycle PCR with stx2 primers. The corresponding melt peaks are shown in Fig 12.

The fluorescent images of the cassette taken by the instrument during the MCA at three different temperatures are shown in Fig 9. With the increasing temperature, the fluorescence sharply drops when the amplified products dissociate causing loss of LC green fluorescence.

### Instrument operation

#### Hot block temperature and pre-biasing

Although the temperature of each block changes less than 2°C during thermal cycling, small, intentional variations occur at appropriate times to achieve the required temperatures in the cassette as it moves from block to block. When the cassette arrives at a block, the cassette and block temperatures equalize to a temperature between their initial temperatures. The equalization temperature depends on their initial temperatures and relative thermal masses. In order to have them equalize to the desired PCR temperature, the block temperature must not be at the PCR temperature before the cassette arrives, but at an appropriate pre-bias temperature. The cassette then settles to the desired PCR temperature.

At thermal equilibrium, some heat escapes from the top of cassette and heat flows up to the cassette from the hot block to maintain the temperature. Small thermal gradients are present in the cassette in this condition. The hot block temperature is held slightly higher than the desired cassette temperature to maintain the cassette at the desired temperature, the working temperature. The user specifies the working temperature and the offset to the pre-bias temperature (offset = pre-bias temperature–working temperature) for each hot block in the PCR settings.

#### Temperature regulation during PCR

The movement of the cassette between blocks is coordinated with the temperature control settings for each hot block and follows a repeating pattern during thermocycling. Each PCR cycle comprises denature, anneal, and extend steps in that order. The cassette takes 7 seconds to move from the denature block to the anneal block at the beginning of the anneal step. Other cassette movements between adjacent blocks take 3 seconds each. For each block, the user specifies the working temperature, the temperature offset (pre-bias temperature—working temperature), and the block time (time the cassette spends at the block including travel time to the block). Cassette movement and the setting of block control temperatures are then automatically controlled by the GelCycler software.

Details of the block temperature control settings and cassette movement are described below for the PCR conditions of 94°C, for 17 seconds, 60°C for 20 seconds, and 72°C for 26 seconds. These conditions result from the working temperature, temperature offset, and block time settings.

**Fig 10:**
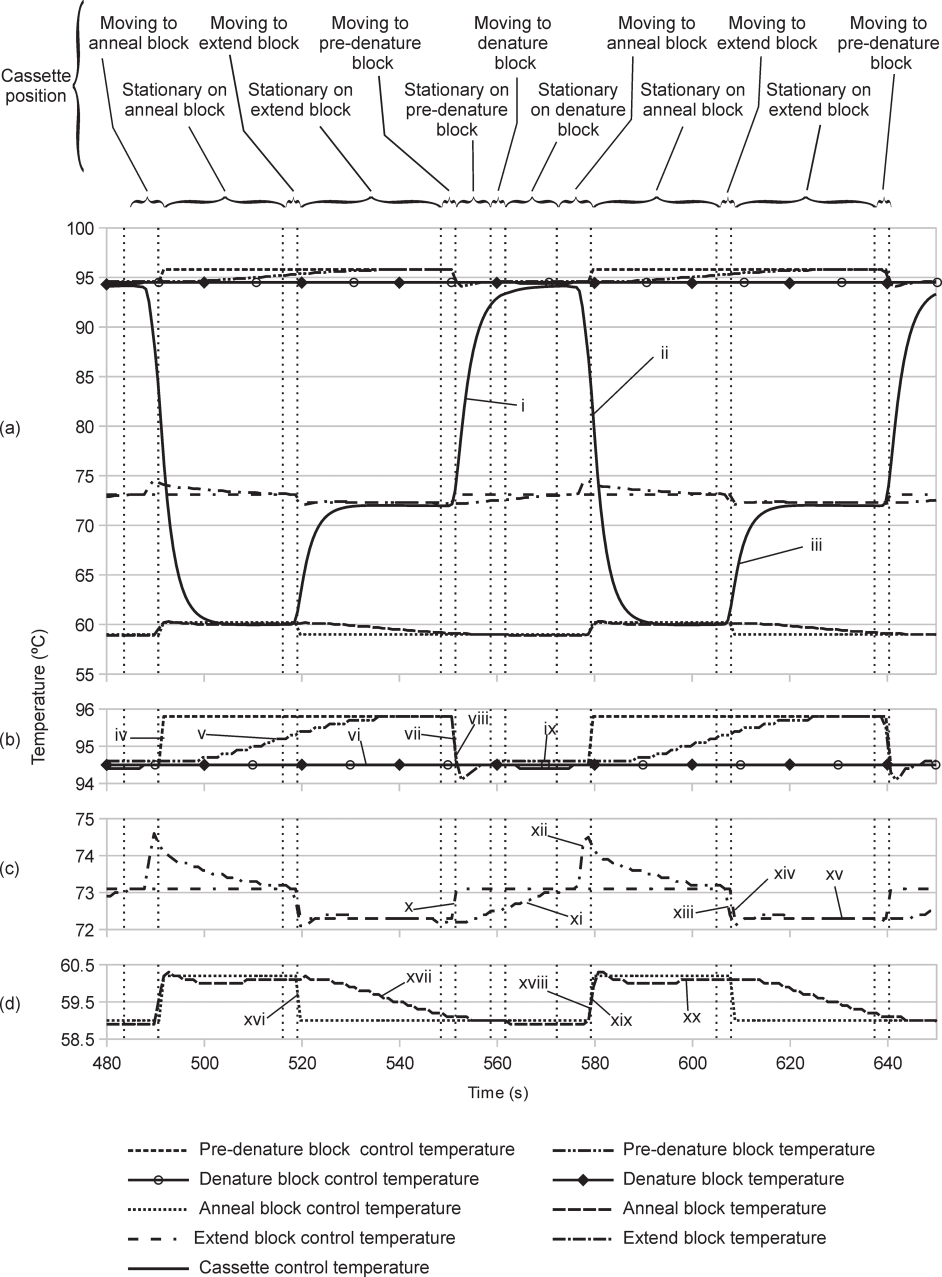
Plot of hot block and cassette temperatures during one cycle of PCR. (a) Pre-denature, denature, anneal and extend block control temperatures and block temperatures, and cassette temperature. (b) Pre-denature and denature block control temperatures and block temperatures. (c) Extend block control temperature and block temperature. (d) Anneal block control temperature and temperature. i) Cassette temperature rise when moved to pre-denature block. ii) Cassette temperature fall when moved to anneal block. iii) Cassette temperature rise when moved to extend block. iv) Pre-denature block control temperature change to pre-bias temperature. v) Slow rise of pre-denature block temperature when the cassette is away from the pre-denature block. vi) Constant denature block control temperature and block temperature. vii) Pre-denature block temperature fall when cassette arrives at the pre-denature block. viii) Pre-denature control temperature change to working temperature when the cassette stops on the pre-denature block. ix) Steady pre-denature block temperature while the cassette rests on the pre-denature block. x) Extend block control temperature change to pre-bias temperature. xi) Slow rise of the extend block temperature when the cassette is away from the extend block. xii) Quick rise in the extend block temperature when the cassette passes over on the way from the denature block to the anneal block. xiii) Extend block temperature fall when the cassette stops on the extend block. xiv) Extend control temperature change to working temperature when the cassette stops on the extend block. xv) Stable extend block temperature while the cassette rests on the extend block. xvi) Anneal block control temperature change to pre-bias temperature. xvii) Slow anneal block temperature fall while the cassette is away from the anneal block. xviii) Anneal block temperature rise when the cassette stops on the anneal block. xix) Anneal block control temperature change to working temperature when the cassette stops on the anneal block xx) Stable anneal block temperature while the cassette rests on the anneal block.

Thermal profiles of the cassette and each block during such a PCR cycle are shown in Fig 10(A) with expanded scales for the block temperatures shown in 10(b), 10(c), and 10(d). The cassette temperature was measured with a small thermistor mounted inside a capillary near the centre of the cassette. Settling time is defined as the time it takes the cassette temperature to change from within 1°C of the initial temperature before a PCR step to within 1°C of its final temperature at the PCR step. The dwell time is the length of time the cassette remains within 1°C of it's final temperature at the PCR step.

#### Denature

The pre-denature and denature blocks are used together to perform the denature step. Prior to the cassette arriving on the blocks, the blocks are prepared by setting their temperatures and allowing them to stabilize. The pre-denature block control temperature is set to it's pre-bias temperature of 95.8°C (Fig 10(B)iv) after the previous denature step and the block temperature slowly rises to that temperature (Fig 10(B)v). After the block temperatures have stabilized, the cassette moves from its previous location (the extend block) to the pre-denature block. Heat flows between the cassette and block and the block temperature rapidly drops to the pre-denature working temperature of 94.5°C (Fig 10(B)vii). When the cassette stops on the pre-denature block, the pre-denature control temperature is changed to its working temperature of 94.5°C (Fig 10(B)viii) so that the block temperature will remain there while the cassette is present. The rapid drop in block temperature is caused by heat exchange with the cassette, and the temperature is then maintained by the temperature control circuit. Ten seconds after moving to the pre-denature block, the cassette moves to the denature block. The pre-denature block control temperature and block temperature remain at 94.5°C (Fig 10(B)ix). The denature block control temperature and block temperature remain at their working temperature of 94.5°C at all times during the PCR cycle. The cassette then rests on the denature block for a period of time giving a dwell time of 17 seconds.

The pre-denature and denature blocks act together for the denature step. When the cassette arrives, its temperature settles (Fig 10(A)i) to 94.15°C (0.15°C above the target denature temperature) with no overshoot beyond that and then drops 0.1°C in the last two seconds of the denature dwell time as the cassette is moving away from the denature block. The cassette temperature takes 9 seconds to settle. The use of two hot blocks for the denature step allows some more advanced temperature control schemes that are not discussed here.

#### Anneal

Prior to the arrival of the cassette on the anneal block, the block is prepared by setting its control temperature to its pre-bias temperature of 59°C (Fig 10(D)xvi) at the end of the previous anneal step and the block temperature slowly falls (Fig 10(D)xvii) to its pre-bias temperature. After the block has reached the pre-bias temperature, the cassette moves to the block. The block temperature rapidly rises (Fig 10(D)xviii) to its working temperature of 60.2°C and the cassette temperature settles (Fig 10(A)ii) to 60°C (the anneal temperature) with less than 0.1°C of overshoot as heat is exchanged between the block and cassette. When the cassette stops, the block control temperature is changed to the working temperature (Fig 10(D)xix) to keep the block temperature steady at the working temperature (Fig 10(D)xx). The cassette temperature takes 10 seconds to settle. The cassette rests on the block for a period of time resulting in a dwell time of 20 seconds.

#### Extend

Prior to the arrival of the cassette on the extend block, the block is prepared by setting its control temperature to its pre-bias temperature of 73.1°C (Fig 10(C)x) at the end of the previous extend step and the block temperature slowly rises (Fig 10(C)xi) to its pre-bias temperature. Note that there is a rapid rise in the block temperature (Fig 10(C)xii) as the cassette moves across the block on the way from the denature block to the anneal block, but the extend block temperature settles back to the pre-bias temperature before the cassette arrives for the extend step. After the block has reached its pre-bias temperature, the cassette moves to the block. The block temperature rapidly falls (Fig 10(C)xiii) to its working temperature of 72.3°C and the cassette temperature settles (Fig 10(A)iii) to 72°C (the extend temperature) with less than 0.1°C of overshoot as heat is exchanged between the block and cassette. When the cassette stops, the block control temperature is changed to the working temperature (Fig 10(C)xiv) to keep the block temperature steady at the working temperature (Fig 10(C)xv). The cassette temperature takes 6 seconds to settle. The cassette dwells on the block for a period of time resulting in a dwell time of 26 seconds. The conditions for the machine warm up before starting the PCR, cassette warm up upon starting the PCR, and the conditions for the first and final PCR cycles are stated below.

#### Initial block heating

Prior to any other actions, the hot blocks are heated to the required temperatures in preparation for the arrival of the cassette. The anneal block control temperature = 59°C, extend block control temperature = 73.1°C, pre-denature block control temperature = 95.8°C, and denature block control temperature = 94.5°C. To start the PCR, the loaded cassette is placed on the cold block when all hot blocks are at the set temperatures.

#### Cassette prewarm

As the PCR is started, the cassette is pre-warmed by moving it to the extend block. When it arrives, the extend block control temperature is set with its working temperature (72.3°C) and the cassette rests there for 10 seconds. It is now ready for the initial denature step.

#### First PCR cycle

After cassette pre-warming, the first PCR cycle begins. It is the same as a standard cycle except for the denature step. The regular pre-denature step is performed. This is followed by a denature step that is lengthened and split into three parts. The block control temperature and block temperature are 94.5°C during the first part, which lasts 60 seconds including the time for the cassette to move to the block. The block control temperature is changed to 94.3°C during the second part, which lasts 60 seconds and to 94.2°C during the third part, which lasts 60 seconds. The cassette does not move during the second and third parts. The control temperature is lowered slightly during this extended denature step to compensate for a slow rise in cassette temperature that would otherwise occur. The denature is followed by regular anneal and extend steps.

#### Final PCR cycle

The final PCR cycle is the same as a regular PCR cycle except that the extend step is 95 seconds longer. The cassette remains on the extend block in order to collect images as described below.

#### Melt curve

The cassette remains on the extend block and the control temperature of the block is set to 68°C, 2°C below the melt curve start temperature. Once the temperature reaches 68°C, the control temperature is set to 87°C, 2°C above the end melt curve temperature. The extend block temperature then rises toward the new control temperature. The cassette temperature will be rising at all times when melt curve photographs are taken. The cassette is illuminated by the LED and images are taken when the block reaches the melt curve start temperature (70°C) and at 0.2°C increments up to the melt curve end temperature (85°C). After the final picture is taken, the cassette is moved to the cooling block and all block control temperatures are returned to their initial settings from the beginning of the GelCycler PCR sequence.

#### Full GelCycler PCR sequence

A full GelCycler PCR sequence proceeds as follows:

- The GelCycler and the computer are switched on.
- An appropriate PCR program is selected.
- The hot blocks warm to their initial temperatures.
- A cassette with hydrated capillaries is loaded into the cassette carrier at the cooling block.
- The automated GelCycler PCR program is started.
- The cassette pre-warm step is performed.
- Thermal cycling is performed by moving the cassette between the heated blocks and the images are taken at the extend step of each cycle.
- The melt curve data is collected for the temperature range specified in the program.
- The cassette returns to the cooling block, where it can be unloaded.

### Functionality of the GelCycler Mark II and aluminum cassettes for detecting targets that identify pathogenic *E*. *coli*

The layout of the heating stage for the new instrument provides additional margin space for a modified (larger) cassette footprint in case a larger number of samples and/or targets is desired for future runs. The present aluminum cassettes hold 50 capillary reaction units, which allows, for example, testing of 8 samples for 5 targets per sample, with the remaining 2 trenches assigned for integrated negative and positive controls. The cassettes have alignment notches so the cassette can only be run in the proper orientation. The aluminum trench construction has two additional advantages in that it blocks any sideways light transfer between adjacent trenches and it enhances the upward redirection (and hence detection) of emitted light from fluorescing amplicons since the cassette surfaces are moderately reflective. Preparation of the capillaries and cassettes for detecting pathogenic *E*. *coli*, details of the *E*. *coli* sample, loading of the sample to the cassette, and PCR/MCA parameters are described below.

#### Capillary preparation

The sequences of the *stx2* primers used to detect shiga toxin-producing *E*. *coli* were described by Huszczynski, et al [21]. One hundred μL gel-PCR reaction mix consisted of 20 μL of 5xPCR buffer [333 mmol/L tris sulfate, pH 8.6, 83 mmol/L (NH4)_2_SO_4_ (Sigma, St. Louis, MO) and 40% sucrose (Sigma)], 30 μL of 40% trehalose (Acros, New Jersey, USA), 2 μL of 100 mmol/L MgCl_2_ (Ambion, USA), 2 μL of 10mmol/L dNTP (Thermo Fisher Sci.), 2 μL of 2% bovine serum albumin (Ambion), 7 μL of 10 μM primer solution (Integrated DNA Technologies, San Diego, CA), 10 μL of 10x LC Green Plus (BioFire, Utah, USA), 6 μL of 20 U/mL Taq polymerase, 10 μL of a 40% acrylamide (Fisher) + 4% bis-acrylamide aqueous solution (N,N-methylene bisacrylamide, Bio-Rad, Hercules, CA), 2 μL of 3% azobis (Wako Bioproducts, Richmond, VA), 1 μL of 10% N,N,N’,N’ tetramethylethylenediamine (Sigma), and water. The mixes were vortexed, centrifuged, and loaded into the capillaries. Polymerization and the desiccation of the gel/PCR mix were completed as previously described [15].

#### Preparation of the cassettes

The cassette was heated to ~ 70°C and each of the 10 trenches was filled with ~ 40 μL of molten wax (Surgipath Paraplast X-tra, Leica Microsystems, Deerfield, IL). The cassette was cooled to room temperature and 5 capillaries with all the gel/PCR reagents desiccated inside were laid in each trench.

#### Sample

A single colony of pathogenic *E*. *coli* ((STEC) ATCC 43895) was suspended in 10 mL TSB (*Tryptic soy broth*, Bacto, Le Pont de Claix, France) and incubated for 18 hr @ 37°C. The final culture (estimated to be about 1.07 x10^9^ bacteria/mL, based on plating) was heated at 90°C for 10 min to inactivate the pathogens. This heat-inactivated culture was then serially diluted in sterile water for delivery to capillary reaction units in cassette PCR.

#### Sample loading

In order to hydrate the gel/PCR reaction mix, 35 μL of sample was loaded to the 5 capillaries in each trench by administering the sample to the capillary at one of the ends. The sample flows through all 5 capillaries by capillary forces [1]. The desiccated gel/reaction mix needs about 10 min to hydrate with the sample.

#### Polymerase chain reaction and melt curve analysis (PCR/MCA)

The GelCycler Mark II is controlled via a laptop computer through a LabVIEW interface. The DNA amplification for the *stx2* target was performed with a pre-denaturation step of 94°C for 3 min, then 35 cycles (unless otherwise specified) of 94°C for 15 sec, 61°C for 20 sec, and 72°C for 20 sec, followed by a final amplification of 72°C for 2 min. At each PCR cycle, a fluorescent image of the cassette is taken at the extension of the PCR cycle. Upon the completion of the PCR, MCA was performed by heating the cassette from 70°C to 85°C and the CCD images were taken at 0.2°C degree intervals.

#### Detection limits for cassette PCR run in the Mark II

To characterize the sensitivity and speed of the Mark II when running cassette PCR, we assembled three separate cassettes with capillary reaction units, loaded them with a calculated number of pathogenic *E*. *coli* from the heat-inactivated culture and then performed PCR at 25, 30 and 35 cycles followed by MCA, for 41, 47 and 53 min reaction times, respectively, to identify the limit of detection for each set of conditions. The above cassettes were tested using the dilutions from an overnight culture of pathogenic *E*. *coli* (estimated to be about 1.07 x10^9^ bacteria/mL, as explained above). Each testing dilution includes a trench holding five replicate capillaries with a published primer set for amplifying *stx2* [21]. Two Mark II instruments were made and tested. In all testing, both gave identical results when run with identical cassettes (not shown).

**Fig 11:**
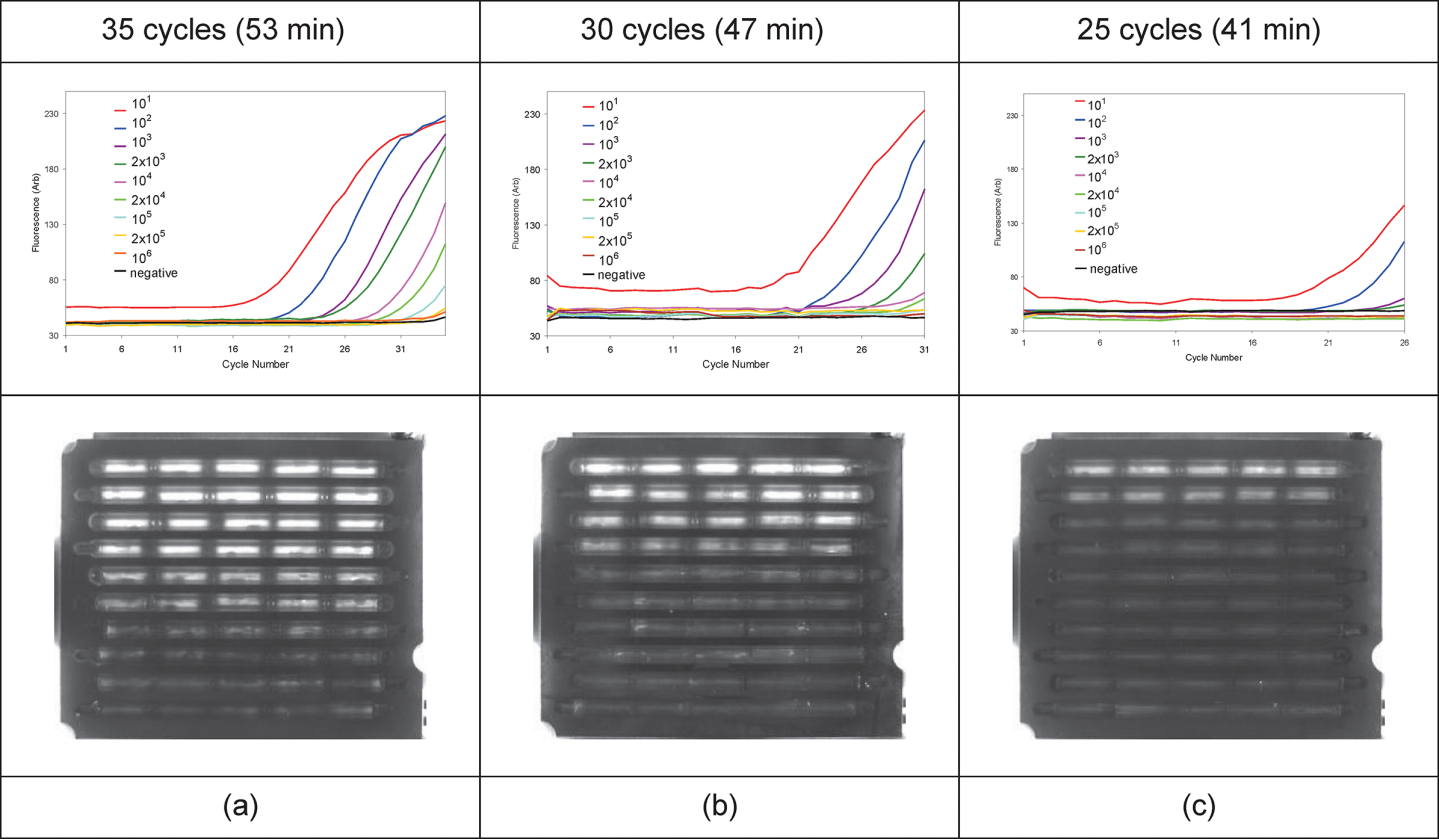
Real-time PCR for the amplification of stx2 gene in diluted overnight culture of *E*. *coli*. PCR was performed for (a) 35, (b) 30, and (c) 25 cycles. The curves show fluorescence amplitude versus number of PCR cycles. The second row shows the CCD images at the 35^th^, 30^th^, and 25^th^ cycle for each PCR, respectively. Capillaries in trenches 1–8 were hydrated with the diluted culture of *E*. *coli* with dilution factors of 10^1^, 10^2^, 10^3^, 2x10^3^, 10^4^, 2x10^4^, 10^5^, 2x10^5^, and 10^6^ respectively. Trench 10 was hydrated with water.

**Fig 12:**
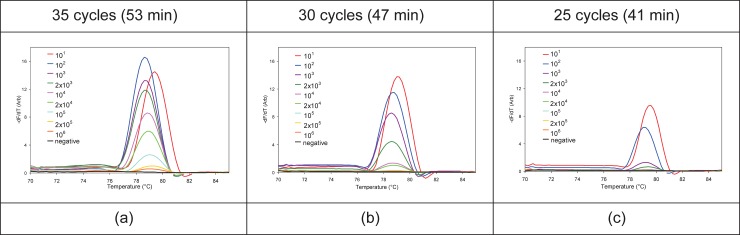
MCA curves for the PCRs performed with (a) 35, (b) 30, and (c) 25 cycles (shown in Fig 11).

**Fig 13:**
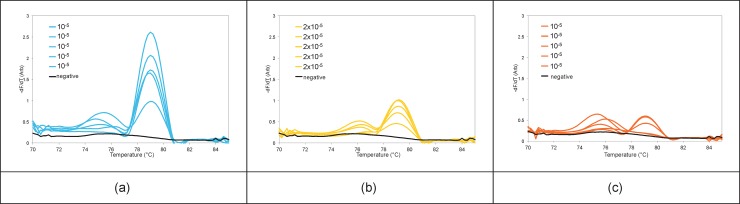
MCA curves of the five capillaries in the entire trench for the three highest dilution factors. (a) 10^5^, (b) 2x10^5^, and (c) 10^6^ for the PCRs performed with 35 cycles (from Fig 11). MCA curve of one of the negative capillaries is plotted to show the baseline.

Fig 11 shows the real-time PCR curves for 35, 30, and 25 cycle PCR reactions, with a series of *E*. *coli* dilutions performed in three different cassettes. The last trench shows the negative control. Each curve represents one capillary out of five from each trench: all 5 replicate capillaries gave comparable results, with the exception of those amplifying *E*. *coli* culture concentrations that were at limiting dilution. MCA curves for the PCR reactions from Fig 11 are shown in Fig 12. Variations in the melt peaks in the same trench at high dilution factors are shown in Fig 13 for the PCRs performed with 35 cycles. Only one of the melt peaks of each of these dilutions are shown in Fig 12(A). Because the serial dilution process suffers from significant variation due to a variety of technical factors, the absolute number of cfu per unit volume is an estimate that for more accuracy requires a fractional analysis to confirm which dilution has a limiting amount of pathogen and thus results in fractional delivery of 1–3 bacteria to each capillary reaction unit. For the series tested here, the greatest dilution, a nominal dilution factor of 10^6^, exhibited a limiting dilution with only 3 out of 5 capillaries as positive after 35 cycles of PCR (Fig 13(C)). This provided an approximate range for dilutions to be tested in the larger fractional analysis reported below. PCRs with 25 cycles had the least sensitivity. By comparison, with 30 and 35 cycles, it is possible to detect calculated culture dilution factors of 10^5^ and 10^6^, respectively.

To determine the level at which culture concentration becomes limiting, indicative of an average distribution of one bacterium per capillary (Poisson distribution), multiple replicate PCR reactions were performed at 35 cycles in GelCycler Mark II, with 45–90 reactions for each culture dilution with higher dilution factors. Approximate culture dilution factors of 1.4x10^5^, 3.3x10^5^, 10x10^5^, and 14x10^5^, were each performed with 45, 90, 90 and 45 replicates, respectively. Each cassette of 45 replicates also had a positive and a negative control. Table 1 shows that dilution factors of 3.3x10^5^, 10x10^5^and 14x10^5^ had limiting numbers of pathogens such that only a minority of the capillaries were positive, roughly fitting the predictions for a Poisson distribution in which the positive capillaries likely received only one bacterium copy. This suggests that cassette PCR reaction as run on the new GelCycler Mark II instruments has sufficient sensitivity to detect in the range of 1 pathogenic bacterium per capillary reaction unit, in 53min (35 cycles), although the peaks were small.

**Table 1:** Detection of 1–3 bacteria using the GelCycler Mark II as determined by a Poisson distribution/fractional analysis of positive capillary reaction units.

Dilution Factor	Number of positives
1.4x10^5^	45/45
3.3x10^5^	78/90
10x10^5^	28/90
14x10^5^	2/45

Currently the Mark II cassette can test 8 samples for 5 different targets with integrated positive and negative controls for each target. GelCycler Mark II PCR reactions are reproducibly faster than in our previously published instrumentation [12–16,19,20], particularly when only low numbers of bacteria are present. Where the original GelCycler was only able to allow direct (un-enriched) detection of about 100 bacteria per reaction in about 2 hours, the improved GelCycler Mark II reliably allows direct detection of about 6 bacteria per reaction in 53’, in a non-fractional un-enriched analysis, a 10 fold improvement in detection sensitivity, and about 2 fold increased speed of the reaction. The aluminum cassettes and the heating stage are sufficiently flexible that larger or smaller heating plates are feasible should this be required by the test panel required, including modifications to allow multiple cassettes to be run simultaneously.

## Conclusions

We found superior performance of the GelCycler Mark II for its sensitivity and speed of the PCR reaction, considerably exceeding that of the original GelCycler reported in previous publications ([12–16,19]). In a fractional analysis, we detect 1 bacteria per reaction unit though the melt peaks were weak. The two GelCycler Mark II systems were made simultaneously and possess identical performance, promising potential for mass commercial manufacture. The machined aluminum cassette used in the new instrument is highly thermally conductive, prevents light bleeding between adjacent trenches, and is reproducible. The design of the cassette can easily be changed to accommodate the required tests per a given sample or the number of samples per given test. The instrument also has the flexibility to allow modifications to simultaneously run more than one cassette. The new GelCycler Mark II along with the ready-to-run new PCR cassette, can reduce detection times of food pathogens presently impacting the food industry.
